# A Series of American Clinical Lectures

**Published:** 1877-04

**Authors:** 


### Books Reviewed.

*A Series of American Clinical Lectures.* Edited by E. C. SEQUIN, M. D. Vol. II.; January to December, 1876. New York: G. P. Putnam's Sons, 182 Fifth Avenue. 1877, pp. 340.

In clearness and thoroughness of detail these twelve lectures are models of clinical instruction. They are pleasant to read, and desirable for the library, being faithful indices of the best American medical practice and opinions of to-day.

Under the head of "The Principle of Physiological Antagonism as Applied to the Treatment of the Febrile State," Dr. Bartholow considers the value of quinia, digitalis, aconite and veratum viride as apyretics. Of salicylic acid he says; It seems in a high degree probable that the anti-anti-pyretie effects are due to its power to arrest the activity of disease and disease-producing organs, and to destroy infectious material and ferments. Of cold baths he says; "Although for the condition of high temperature, especially for hyper-pyrexia cold baths, 96° to 60° F., must be regarded as the most appropriate remedy; we are nevertheless in many inflammatory afflictions restricted to the use of anti-pyretic medicines."

As a conclusion, "When the state of pyrexia is the most important element in the morbid complexus, cold baths, quinia and digitals are the remedies to be employed. In the fever of inflamation, is the action of the heart vigorous and the arterial tension high? aconite and veratum viride are indicated. Is the action of the heart feeble and the tension of the vessels low? quinia and digitalis are more appropriate. Is the fever due to putrid ferments, to disease-producing organisms? quinia and salicylic acid are required. Dr. Jewell speaks of a lesion of nutrition in "certain forms of morbid nervous sensibility," calling for rest, and nutritive material; also of a "loss of balance between waste and repair." He says; "In ninety-nine cases out of a hundred of nervous disease, you will have to meet with this lesion of nutrition caused by too much action and too little rest, or by want of nutritive matter in the blood." Farther on; "But I am safe in saying that the value and necessity of rest, physical, mental and emotional, in the various forms of neurasthenia is only beginning to be understood by the profession." Rest he defines as cessation or change of activity. Among other remedies he mentions the acid phosphate of lime as an excellent appetizer. He also says: "You will find electricity intelligently and faithfully used one of your most effective agents in nervous disease. As a sedative, the prolonged use of ergotine, in doses of from two to five grains is recommended, combined with one-fourth to one-sixth of a grain of digitalis, if the circulation is fluctuating.

Dr. Seguin, in his lecture on "Melancholia at Home," says; "Opium, cannabis, indica and alcohol often make melancholic patients sleep very well. They improve the nutrition of the brain, render the circulation in it more active and thus expedite a cure. Opium and cannabis I give in pillular form pushing the opium up to the point of slight narcosis and seldom giving more than a grain and a half of cannabis in twenty-four hours." Aside from these agents for procuring sleep, he choses from the "restoratives" of Headland to improve nuitrition, seeks to remove the cause of the prostration, whatever that may be, and the patient must have three good meals every twenty-four hours, of meat, eggs, etc., and unbroken sleep at night, with a cheerful moral atmosphere by day. He says; "Galvanization of the spinal cord, and of the cervical sympathetic have been used in Europe and in this country with apparent success. I have great faith in the efficacy of amusement and employment in the treatment of insanity in general, and of melancholia more particularly. If you are not faithfully and actively aided by the patient's friends, or by nurses, in carrying out the moral treatment, medication will prove quite useless." Dr. Delafield calls the stomach pump to his aid in cases of dyspepsia of long standing, characterized by pain in the stomach and vomiting, using it once a day, at most, and three hours after the taking of a meal of solid food. For functional derangement of the small intestine, another form of dyspepsia, he says; "The drugs indicated are cubebs, ipecac and asafœtida; cubebs, ten grains of the powder or twenty minims of the tincture; ipecac, one-eighth of a grain, increased gradually to four grains, asafœtida in four-grain sugar-coated pills, or the compound Galbanum pill. The great majority of cases of dyspepsia, coming to this clinic are cases of liver dyspepsia." These he divides into two classes, the fat and the lean. Exercise in the open air is prescribed for both, violent muscular exercise for the former, riding and short walks for the latter. Constipation, existing in both cases, is to be relieved in the former, by a mild laxative, as the dinner pill, in the latter by rhubarb, strychina, podophyllin, aloes, etc.; mineral acids to improve the appetite.

Dr Noyes devotes sixty pages to a consideration of the diseases of the eye, and it is an admirable lecture.

Dr. Sands urges the performance of laryngotomy, or tracheotomy, when suffocation is impending, whether there are grounds for hope of the ultimate recovery of the patient or not; as, if well and carefully done, it makes even the small remaining portion of life more comfortable, and it is often impossible to say who will recover, and who may not. He proposes to have the patient anæsthetized, usually employing ether. "Methods of Examining the Upper Air Passages" are ably given by Dr. Lefferts. He describes in minute detail every step of the operation. His language is most concise, and direct, and we cannot see how instruction could be better or more forcibly given. The hypertrophied prostate, and its treatment, are well and carefully considered by Dr. Weir.

Dr. Pooley, in discoursing of "Congenital Phimosis," says; "I have come to take what many will consider extreme ground on the subject of nocturnal enuresis. I regard this affection, in boys, as almost always the result of phimosis. Not necessarily of extreme phimosis, but quite often of that moderate degree of it, which may almost be considered the normal condition in little children." His remedy is circumcision. Of hare lip he says: "In the operation itself probably the most important step is the thorough detachment of the lip from its osseous and other attachments, so that the edges, when freshened, may come completely and easily together, without the least strain." He uses the simple interrupted suture, and leaves the lip uncovered.

Of "Spinal Irritation," Dr. Hammond says: "Its symptoms are more certainly referable to anæmia than to any other condition. Those medicines, such as strychnine, phosphorus and picrotoxine, which increase the amount of blood in the cord, are the remedies which are most effectual in combating the disease, while others, such as ergot and belladonna, the action of which is to diminish the amount of intra-spinal blood, invariably aggravate the symptoms. Dr. Jewell believes that spinal derangement is the result of visceral trouble. Dr. Hammond believes that visceral derangements are more frequently the *result* of *spinal anæmia.* He says: "Opium is a remedy of great power in spinal irritation. The entire drug is, I think, better than any of its constituents. A pill of a grain may be taken morning and night. Alcohol, in some form or other, is an article of food which cannot well be done without. Among medical means, for relief of vomiting, I have never used anything comparable to valerianate of caffeine, which may be given in doses of from three to five grains, as often as it appears to be required. In a very obstinate case which I saw recently, in consultation with Dr. Whybrew, every noted medicine had been tried without effect, when a couple of doses of valerianate of caffeine arrested the vomiting."

The Treatment of Eczema is by Dr. Taylor, of Charity Hospital, New York. He uses frequent applications of very hot water in dry eczema, glycerine and water in the intervals being applied on linen lint. In moist eczema the hot water, he says, does harm, though a few ablutions may be used to relieve burning pruritus in the most severe stage. Glycerine and black wash on lint is a favorite application. In both varieties an alkaline cathartic treatment is used. The lead and opium wash, with a little glycerine, is also recommended for the moist varieties. He says for dry eczema the remedy which I use most frequently here in the clinic is Anderson's powder, as follows :

R.

Pulv. Starch, oz. j.
Oxide of Zinc, oz. ss.
Pulv. Camphor, oz. iss.

M.

This must be made into a perfectly impalpable powder and freqently and thickly dusted over the affected skin, or rubbed into fine linen lint and applied. For eczema capitis, an oxide of zinc and mercury ointment is used after the scabs have been loosened by glycerine, and gentle manipulation with a comb, and removed. Bismuth may be used in the place of the oxide of zinc and camphor added to lessen pruritas. The child's diet is to be carefully regulated, milk being given it for food in preference to anything else. A little bread and meat both may also be used.

"Peripheral Paralysis," by Dr. Miles, of Baltimore, closes the volume. He says : "The remedy I have found most useful in peripheral paralysis, the remedy par excellence, is electricity, either the iuduced or galvanic current. While many regard it a matter of indifference which of these two sorts of electricity we apply I must say that in the great majority of cases I give the decided preference to the galvanic or constant current, though I advise you not to use either exclusively, to the neglect of the other, but seek to combine their employment in your treatment." It is impossiblo in so brief a review to do more than indicate the general scope and direction of this work. It should be read to be properly appreciated.

